# Noninvasive Electroencephalogram Based Control of a Robotic Arm for Reach and Grasp Tasks

**DOI:** 10.1038/srep38565

**Published:** 2016-12-14

**Authors:** Jianjun Meng, Shuying Zhang, Angeliki Bekyo, Jaron Olsoe, Bryan Baxter, Bin He

**Affiliations:** 1 Department of Biomedical Engineering, University of Minnesota, Minneapolis, MN, USA; 2 Institute for Engineering in Medicine, University of Minnesota, Minneapolis, MN, USA.

## Abstract

Brain-computer interface (BCI) technologies aim to provide a bridge between the human brain and external devices. Prior research using non-invasive BCI to control virtual objects, such as computer cursors and virtual helicopters, and real-world objects, such as wheelchairs and quadcopters, has demonstrated the promise of BCI technologies. However, controlling a robotic arm to complete reach-and-grasp tasks efficiently using non-invasive BCI has yet to be shown. In this study, we found that a group of 13 human subjects could willingly modulate brain activity to control a robotic arm with high accuracy for performing tasks requiring multiple degrees of freedom by combination of two sequential low dimensional controls. Subjects were able to effectively control reaching of the robotic arm through modulation of their brain rhythms within the span of only a few training sessions and maintained the ability to control the robotic arm over multiple months. Our results demonstrate the viability of human operation of prosthetic limbs using non-invasive BCI technology.

Individuals who suffer from severe neuromuscular disorders or damage to the motor system, including muscular dystrophy, brain stem stroke, and spinal cord injuries, frequently lose the ability to freely move and control their muscles. However, most of these individuals retain the ability to produce motor function-related neural activities similar to healthy subjects, as the brain areas orchestrating movement often retain function despite some loss of peripheral motor control. Brain-computer interfaces (BCIs) are a class of emerging technology that aim to directly bridge the brain and the outside world^1^. One of the ultimate goals for BCIs is to enable the anthropomorphic movement of a highly dexterous prosthetic limb, or exoskeleton as an assistive device, by deciphering a patient’s brain activity in real time. A major challenge for emulating brain-to-limb control is building a complex and robust interface to coordinate the high number of degrees-of-freedom (DOF) needed to achieve anthropomorphic control. This challenge is amplified when using non-invasive measurements to replace the delicate control between the brain and body. Over the past few decades, BCI technologies have been developed using several different input signals. BCIs using intra-cortically implanted electrode arrays can measure the activity of tens to hundreds of neurons in movement-related cortical areas. These arrays cover several square millimeters, have a high signal-to-noise ratio (SNR), and have facilitated promising research results for the control of robotic arms or patient’s own arm through neuromuscular electrical stimulation^2^,^3^,^4^,^5^,^6^,^7^,^8^. However, these invasive approaches face the risk of post-surgery complications and infections, and the challenge of maintaining stable chronic recordings, which might limit broad use in the patient populations^9^. For noninvasive EEG, no surgery is needed and little time is required to place the scalp electrodes. Noninvasive EEG based BCI could potentially serve the needs of a large population^10^.

Control of a robotic arm with non-invasive BCI certainly provides a desirable alternative, but prior to this study it has not been shown that such non-invasive systems could achieve proficient multi-dimensional control of a robotic arm to reach and grasp an object in three-dimensional (3D) space. The concept of machine control via non-invasive EEG has been proposed in previous research, and explored in successful offline analyses or online cases including control of a virtual object^11^,^12^,^13^, real objects such as wheelchair, quadcopter^14^,^15^, and various other rehabilitation and assistive devices^16^,^17^. To the best of our knowledge, few research groups have attempted control of a prosthetic or a robotic arm using scalp EEG based BCIs. A variety of control signals, including sensorimotor rhythms^18^, steady state visual evoked potentials^19^,^20^, hybrid systems^21^, real movement or attempted movement^22^,^23^, have been used for these initial studies to control the robotic or prosthetic arm. Such previous efforts have primarily constrained the BCI control system to be discrete in one dimension or a plane without exploring the full possibility of controls in three-dimensional space. In the present study, we examine the possibility of using non-invasive motor imagination based BCI for control of a robotic arm to complete reach and automatic grasp tasks in three dimensions, with the following question in mind: is non-invasive BCI capable of providing sufficient precision and efficiency to control a robotic arm in a 3D environment to complete meaningful reach-and-grasp and complex tasks?

To address the above question, we designed a series of experiments with progressively increasing task difficulty, and recruited a group of healthy human subjects to use non-invasive BCI to control a robotic arm for performing complex reach-and-grasp tasks. The reach-and-grasp task was divided into two stages: first, the subject was to guide the cursor/robotic arm within a two-dimensional (2D) plane to a region above a target object within 3D space and hover over it; second, if the subject selected the correct object s/he was then to guide the robotic arm down in the third dimension to grasp the object. This two-step sequential experimental design effectively reduced the number of DOF that the BCI needed to interpret, while still allowing participants to grasp an object in 3D space. This simplifies the process of grasping an object in 3D space by performing reach-and-grasp tasks sequentially, with a tradeoff of slightly increasing the time required to perform the complete task. 13 healthy subjects demonstrated the capability of learning to modulate their brain rhythms to control a robotic arm using our non-invasive system with two-stage control. Subjects efficiently learned to manipulate a robotic arm to grasp and move objects randomly located in a restricted 3D space, and maintained their control ability over multiple sessions across 2–3 months.

## Results

### Brain-control tasks

**Figure 1:**
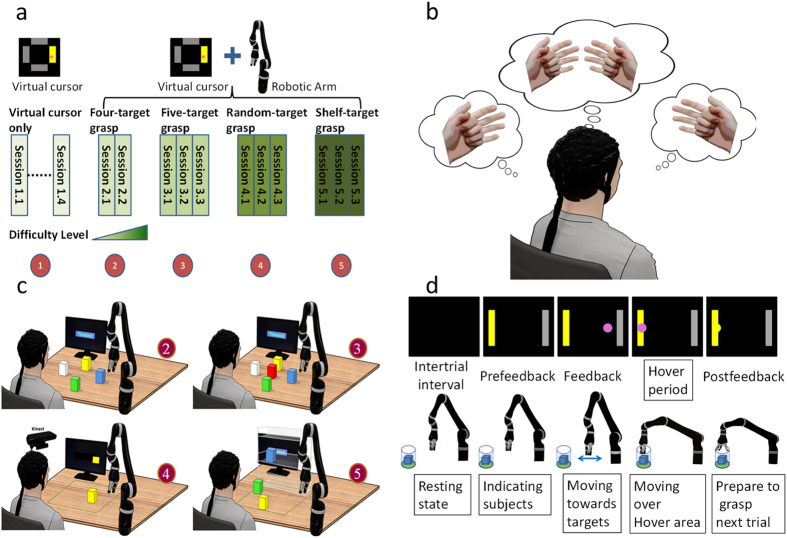
Experiment setup and task progression. (**a)** Overview of experimental sessions for each participant. There were five stages of experiments with increasing level of difficulty, where each stage included two to four sessions of the same experimental paradigm. (**b)** Motor imagery tasks were used to drive two dimensional virtual cursor or robotic arm movement. The imagination of left hand, right hand, both hands, and relaxation corresponds to the respective left, right, up, and down movement of the robotic arm and virtual cursor. (**c)** Overview of tasks for experiment stages two through five. Experiment stage two (four-target grasp): Grasping one of the four fixed targets. Experiment stage three (five-target grasp): Grasping one of the five fixed targets. Experiment stage four (random-target grasp): Grasping a randomly located target. Experiment stage five (shelf-target grasp): Moving one target from the table onto the shelf. (**d**) Trial structure of a single trial task. First, there is a short period of inter-trial interval between two separate trials. After that, the target is displayed on the screen for three seconds during the prefeedback period and is followed by a moving pink cursor and robotic arm in the respective workspaces during the feedback period. If the robotic arm remained within the predefined radius above the designated block for 2 seconds, the hover period would be complete and the task would progress to the step of grasping in the reach-and-grasp sequence (otherwise the step is timeout after 12 seconds and a new trial begins). At this point, the computer would recognize that the robotic arm was meant to stop and grasp the target. The robotic arm would open the fingers and be prepared to finish the grasping sequence during the next trial (step) if the subject controls the robotic arm to move towards the block correctly.

13 healthy human subjects were recruited to perform a series of experimental tasks across 8–15 sessions with increasing task difficulty according to Fig. 1a. Each session consisted of 10–12 runs with break time in between; each run was 5–10 minutes long and varied among individuals and task levels. There were a total of five chronological stages across the 15 sessions: virtual cursor only, four-target grasp, five-target grasp, random-target grasp and shelf-target grasp (see Fig. 1c for visualization). The subjects were instructed to imagine movement of their left hand, right hand, both hands, or relaxation of both hands to control the left, right, up and down (forward or backward) cursor and robotic arm movement, respectively (Fig. 1b). Through the instructed imagination they learned to modulate their sensorimotor rhythm amplitude in the upper mu (10–14 Hz) frequency band. The power of mu rhythm was then linearly mapped to control the velocity of cursor and robotic arm movement (**Methods**). Subjects were instructed to perform kinesthetic motor imagination in the first person perspective^24^.

### Training performance of virtual cursor control

**Figure 2:**
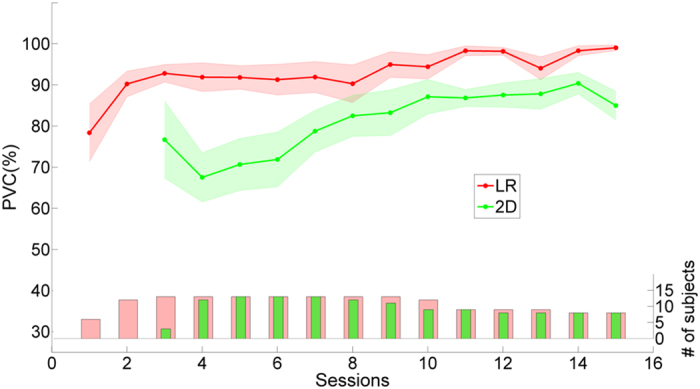
Overall learning process of virtual cursor control. Learning processes (PVC) of 1D LR and 2D cursor movement for all subjects across all sessions. Average PVC for LR and 2D are highlighted by the red and green lines, respectively. The standard errors of the mean (SEM) are indicated by the shaded regions alongside the two lines. Since not all subjects participated in all 15 sessions, the number of subjects included in each 1D LR and 2D session which are arranged chronologically are indicated, respectively, by the red and green bar plots in the lower part of the figure.

In each session there was at least one run of one-dimensional (1D) left-right (LR) cursor control, and each subject participated in up to 15 sessions. The average percent valid correct (PVC) of all the subjects for 1D LR cursor movement control across all sessions is displayed as the red line in Fig. 2. The PVC is defined as the ratio of the correct target hit versus all of the valid outcomes. Thus, invalid outcomes corresponding to those trials when neither the correct nor an incorrect target was hit are excluded in the calculation of PVC. At the first session, the average PVC for LR was 78.4 ± 7.0% and rose to 90.2 ± 3.1% at the second session. At later sessions near the end of training, the average PVC exceeds 95% for 1D LR control.

For each subject, there were two to four runs of 2D cursor control task (left-right or up-down) in each session. Subjects could intentionally control a virtual cursor displayed on the monitor to move freely in a restricted square area for this task. The average PVC for 2D cursor control of all subjects across all sessions is displayed as the green line in Fig. 2. The number of subjects in each session is shown in the green bar plot below. Subjects were required to demonstrate proficiency in 1D LR cursor control prior to progressing to 2D cursor control. Therefore there were several sessions at the beginning of training in which subjects did not perform the 2D virtual cursor control task. The average PVC was 77.0 ± 9.3% at the first session, dropped slightly to 67.5 ± 6.0% at the second session, and then increased mainly across the remainder of the training. The drop in performance after the first 2D session is most likely due to an increase in the number of subjects from the first to the second session. The average PVC increases above 85% after seven sessions of practice. Theoretically, chance level performance is 25% for 2D cursor control and 50% for 1D LR cursor control. Overall, there is an upward trend for both 1D LR and 2D cursor control across time at the group level.

### Event related de/synchrozination maps of 2D virtual cursor control

**Figure 3:**
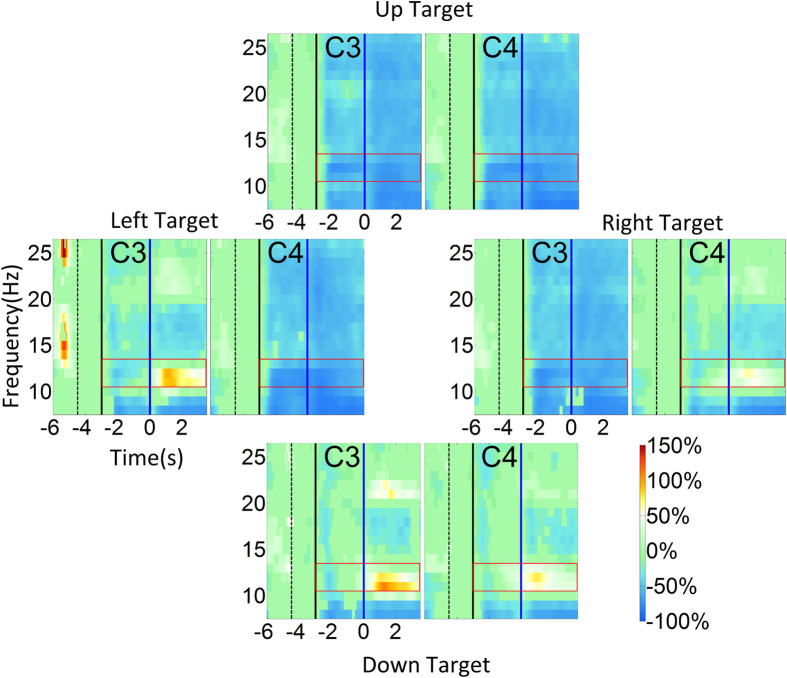
Event related desychronization (ERD)/event related synchronization (ERS) maps of 2D virtual cursor movement. ERD/ERS maps of left, right, up and down target trials for electrodes C3 and C4. In each subplot the horizontal axis indicates the time (seconds); the vertical solid black line denotes when the target appeared, and the vertical solid blue line indicates when cursor control began. The period between the black dashed line and the black solid line shows the baseline period that was used to calculate the ERD/ERS. Only significant changes of ERD/ERS activity quantified by a bootstrap resampling method (see method) were shown here. The 8–26 Hz frequency band is indicated in the vertical axis. The red rectangle centered at 12 Hz (3 Hz bin width) highlights the mu band rhythmic activity starting from the appearance of the target and ending at 3.5 seconds after the cursor began to move. The target appeared at −3 seconds and the virtual cursor control began at 0 seconds. The baseline was selected as −4.5 seconds to −3 seconds during which the screen was black and the subject was instructed to remain in an idle state.

Figure 3 shows the group-level event related desychronization (ERD)/event related synchronization (ERS) maps averaged across all subjects and all sessions for the 2D cursor control task. The subplots display activity from the C3 and C4 electrodes, located over the left and right motor cortex, respectively. Clear contralateral ERD was evident during the right and left target trials when the target appeared and the subject performed the unilateral hand motor imagination (Fig. 3 left target and right target). This ERD was also accompanied by a statistically significant ipsilateral ERS when the cursor began to move. Bilateral ERD was apparent when the subject performed the bilateral hand motor imagination for the up target (Fig. 3 up target), and bilateral ERS was apparent when the subject relaxed for the down target (Fig. 3 down target). Whereas the ERD was apparent both when the target was presented and when the cursor was being moved by the subject, the ERS was usually only significant after the cursor began to move.

### Event related de/synchrozination maps of robotic control

**Figure 4:**
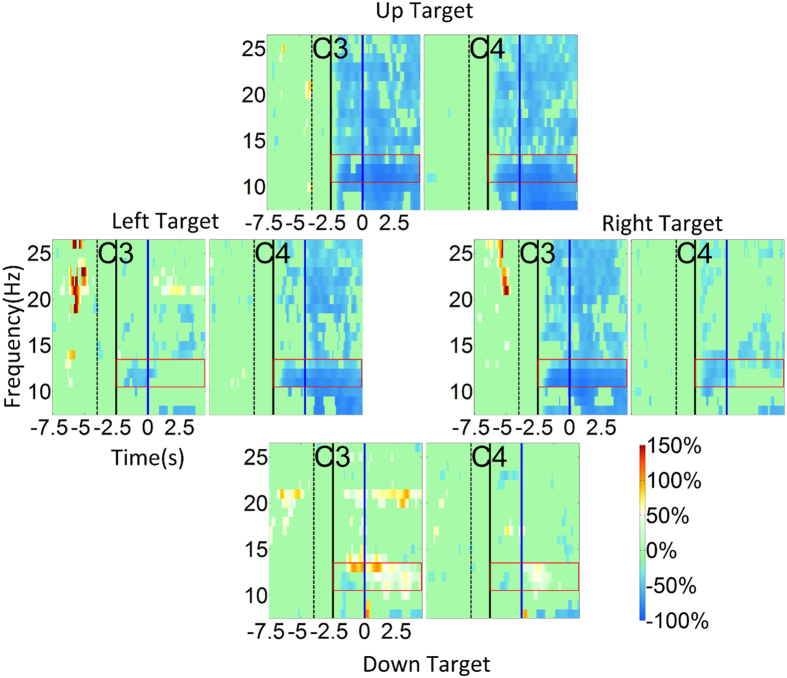
Event related desychronization (ERD)/event related synchronization (ERS) maps of the fixed four target grasping task. ERD/ERS maps of moving towards the left, right, up and down targets for electrodes C3 and C4. The target appeared at −2.5 seconds and the robotic arm began to move at 0 seconds. The baseline was selected as −4 seconds to −2.5 seconds during which the robotic arm was stationary, the screen was black, and the subject was instructed to remain in an idle state.

Figure 4 shows the similar group-level ERD/ERS maps averaged across all subjects and all sessions (four-target grasp task). In general, the plots display similar contralateral ERD for the unilateral hand motor imagination (Fig. 4 left target and right target), and bilateral ERD and ERS for the bilateral hand motor imagination and relaxation tasks (Fig. 4 up target and down target), respectively. Note that there was no strong ipisilateral ERS for the unilateral hand motor imagination task. Additionally, the bilateral ERS was also not as strong as the virtual cursor control counterpart.

### Performance for grasping of fixed four and five targets task

**Figure 5:**
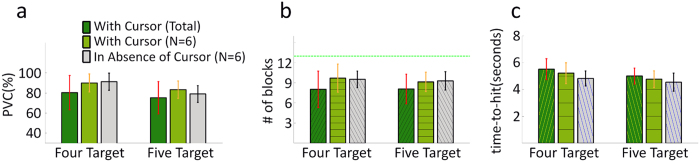
Grasping performance of the four-target and five-target grasp tasks in the presence and absence of the accompanying cursor movement. (**a**) Group average PVC and one standard deviation for the four-target and five-target grasp tasks. The leftmost bar for each task indicates the PVC of the original 13 subjects. The right two bars compare the PVC of the subset of six subjects who participated in additional sessions both with and without the cursor present. (**b**) Average number of blocks grasped in each run of the four-target and five-target grasp tasks for all subjects and all sessions, as well as the subset of six subjects. The green line shows the ideal maximum number of blocks (13 blocks) that can be grasped in each run. (**c**) Average single-trial time-to-hit target for all subjects and all sessions, as well as the subset of six subjects. The feedback duration when the robotic arm moved to complete the individual steps of the reach-and-grasp sequence was denoted as the single-trial time-to-hit.

Figure 5 displays the results for the second and third stage of BCI training, where the subjects were instructed to perform a grasping task with a robotic arm. In these stages, virtual targets and the virtual cursor movement accompanied the robotic arm movement on the computer screen. In addition to the five stages of experiments, 6 of the 13 subjects were able to participate in another three sessions of experiments for controlling the robotic arm in the absence of the virtual cursor. Note that it took two steps for the subjects to pick up the correct block within two separate trials. These two steps included a first step in which the robotic arm had to hover over the center of the specified block for 2 seconds, and a subsequent step in which the robotic arm had to move downward to grasp the block (see Supplementary Figures 2 and 3 for segments of EEG signals and scalp topographies associated with movement trajectories for examples of grasping four different blocks).

The average PVC values across all subjects and all sessions for the four-target and five-target grasp tasks are shown in Fig. 5a. The average PVC for the four-target grasp task increased from 77.8 ± 18.1% in the first session to 82.8 ± 16.3% in the second session, resulting in an average of 80.3% ± 17.0% for the two sessions (Fig. 5a, dark green bar in the column of four targets). The average PVC across the six of the participants for the four-target grasp task sessions in the absence of the virtual cursor was 90.1% ± 7.7% (Fig. 5a, gray bar in the column of four targets). The same six subjects’ average PVC for the four-target grasp task with the virtual cursor across sessions was 89.9% ± 8.9% and is separately displayed as a light green bar in Fig. 5a. A Wilcoxon signed-rank test was applied to compare the performance of the six participants with and without the virtual cursor. There was no significant difference (p > 0.05) between the two conditions when controlling the robotic arm in the four-target grasp task.

Similarly, the average PVC across all participants for the five-target grasp task increased from 74.5 ± 17.3% in the first session to 84.9 ± 6.6% in the third session and resulted in an average of 77.9% ± 14.7% across the three sessions (Fig. 5a, dark green bar in the column of five targets). The average PVC of those six participants for the five-target grasp task in the absence of the virtual cursor across sessions is 79.0% ± 8.3% (Fig. 5a, gray bar in the column of five targets). The same six subjects’ average PVC for the five-target grasp task with the virtual cursor across sessions is 85.1% ± 8.0% and is also separately shown as the light green bar in the column of five targets. The results of a Wilcoxon signed-rank test (p = 0.031) for PVC indicates that there is a significant difference between the two conditions when controlling the robotic arm in the five-target grasp task.

The maximum number of targets that could be grasped in each run was 13, which is highlighted with a green horizontal line in Fig. 5b. The average number and standard deviation (SD) of targets grasped by all subjects in each run across sessions is 8.0 ± 2.7 and 8.4 ± 2.1, respectively, for the four-target and five-target grasp tasks. For the subset of six subjects, the results of a Wilcoxon signed-rank test for the average number of targets grasped indicates no significant difference between conditions with and without the virtual cursor for both the four-target (p > 0.05) and five-target (p > 0.05) grasp tasks. For all subjects, it took on average 5.5 ± 0.8 s and 5.0 ± 0.6 s (Fig. 5c) to complete the individual steps (trials) required to complete the reach-and-grasp sequence in the four-target and five-target grasp tasks, respectively. Similarly, the results of Wilcoxon signed-rank tests for the average duration of step completion indicates no significant difference between experiments with and without the virtual cursor for both the four-target (p > 0.05) and five-target (p > 0.05) grasp tasks. On average, it took 27.1 ± 3.7 s to grasp one block; this time included the inter-trial intervals, prefeedback periods, feedback periods and postfeedback periods in the reach-and-grasp sequence.

### Grasping performance of randomly located targets on a plane

**Figure 6:**
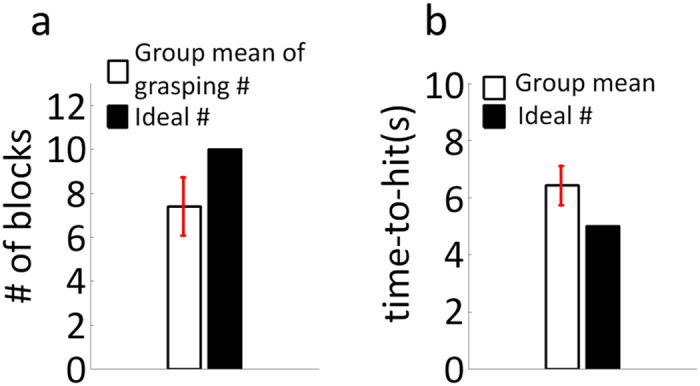
Grasping performance of randomly located targets. **(a**) Average number of targets grasped per run for all subjects and all sessions versus the ideal number of targets that could be grasped per run (10 targets). (**b**) Average single-trial time-to-hit of EEG robotic arm control compared to the ideal time-to-hit of the robotic arm control.

Figure 6 illustrates the results of the fourth stage of robotic arm control. The target block in this stage was randomly placed in the square workspace instead of at fixed positions, as was done in the four-target and five-target grasp tasks. In this stage, subjects had to control the robotic arm to hover above the target block for 2 seconds. Theoretically, a maximum of 10 blocks could be grasped in each run (shown as the black bar in Fig. 6a). The average number of blocks grasped by participants per run was 7.4 ± 1.3, shown as the white bar in Fig. 6a. The number of target blocks grasped varied across subjects and runs depending on the subjects’ ability. Some subjects dropped out of the study before participating in this stage, resulting in a total of ten subjects for this stage. It took subjects on average 6.4 ± 0.7 seconds to finish each single-trial step in the grasping sequence (Fig. 6b), and took 30.5 ± 4.1 s on average to grasp one block for this paradigm. Subjects’ EEG control was compared with the ideal completion time, defined as the shortest time it would take the robotic arm to complete each single-trial step in the grasping sequence with no path redundancy. Experimental design including the hover period as well as physical limitations of the robotic arm resulted in a minimum time of 5 seconds (black bar in Fig. 6b).

**Figure 7:**
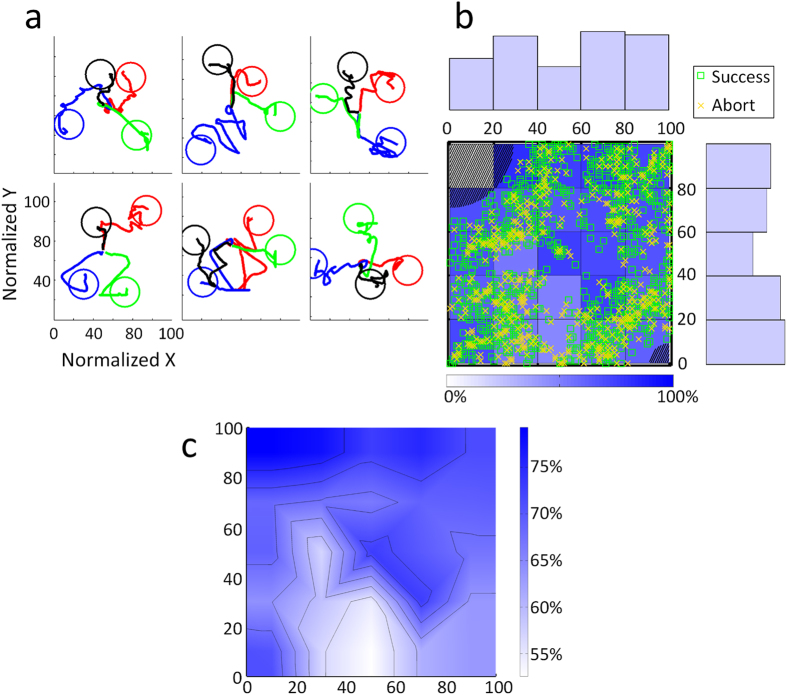
Example trajectories and the distribution of successful grasping trials for randomly located targets. (**a**) 24 example trajectories from six different subjects (four each) for grasping random targets located in the four quadrants. The circles indicate the hover area for the randomly placed targets. (**b**) The distribution of successful and unsuccessful grasping within the workspace. The histograms above and to the right of the plot indicate how often the target was placed in that area of the workspace. (**c)** Topography of successful grasping rate within the workspace.

The movement trajectories of the robotic arm for the random-target grasp task are shown in Fig. 7a for 6 different subjects. Different colors are used to discriminate different targets in the four distinct quadrants. Some of the trajectories were fairly direct to the hover area (the circle) while others might move in and out of the hover area multiple times before finally moving into the area for the required 2 seconds. The group-level distribution of successful grasping for the randomly located blocks is shown in Fig. 7b (see Supplementary Figure 4 for four individual cases of target distribution and successfully grasped blocks). Here, the successful grasping rate was defined as the ratio between the number of successfully grasped blocks and the sum of successful grasping and abort trials. The top left and bottom right portions of the workspace (greyed region) were inaccessible due to singularity problems of the arm. The marginal distributions reveal how often the target was placed in that area. The target was equally distributed among the four quadrants of the workspace and was quasi-uniformly distributed among the whole area, excluding the regions unreachable by the robotic arm. The plot in Fig. 7b shows that the successful grasping rate is higher than 62.2% across the accessible workspace except for some areas near the bottom center areas (light blue areas). For those regions, the median success rate is 53.1% and the minimum is 47.2%. This indicates that subjects could not move to the bottom center area as efficiently as other areas, although they could still successfully control the arm to grasp the targets located in that area. Figure 7c shows the interpolated topography of successful grasping averaged across all subjects and all sessions of the random target task.

### Performance of moving targets from table to shelf

**Figure 8:**
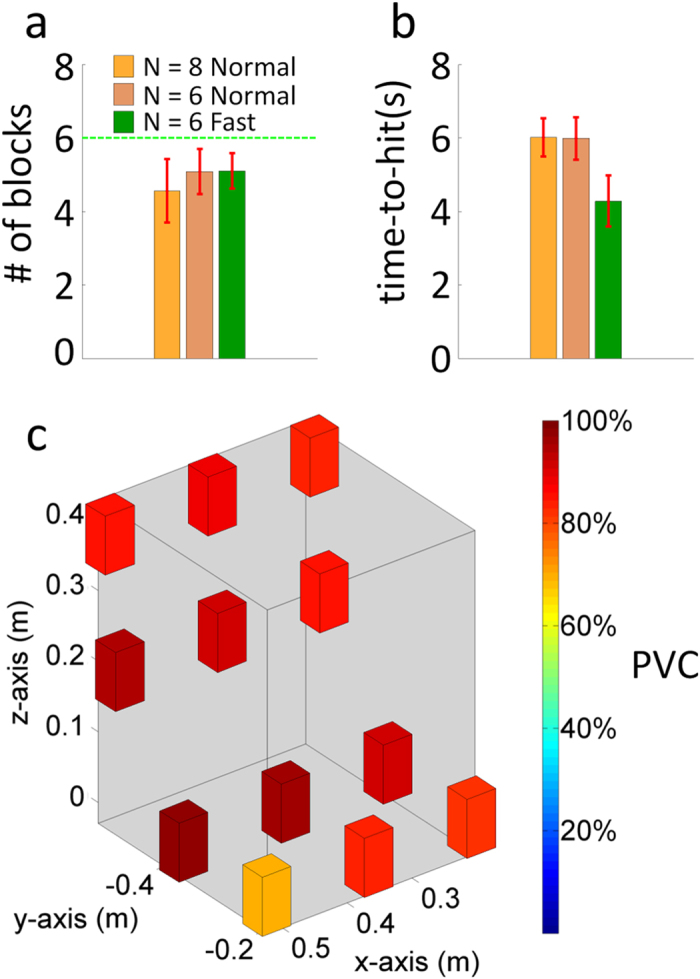
Grasping performance of the shelf-target grasp stage. (**a**) Average number of targets grasped in each run for the original 8 subjects (orange bar) and the subset of 6 subjects who participated in three extra sessions. These extra three sessions involved controlling the robotic arm with the initial normal speed (shelf-target grasp) and an increased speed of movement (fast-shelf-target grasp). The green line shows the ideal number of blocks (6 blocks) that can be reached in a single run. (**b**) Average single-trial time-to-hit and standard deviation for all of the original 8 subjects and the 6 subjects who participated in both the shelf-target grasp and fast-shelf-target graps tasks. (Examples of robotic hand trajectories during the feedback period are shown as blue, yellow, red and green lines in Supplementary Figure 5). (**c**) Distribution of PVC for moving targets from a table onto a shelf. Average PVC of reach-and-grasp for the blocks on the table (x-y plane) and average PVC of reach-and-release for the blocks onto the shelf (x-z plane) are shown.

In the fifth stage, subjects were required to move one of the three blocks from the table to a specified location on a shelf (refer to **Brain-control tasks**). In order to move a block successfully the participants had to finish each of the four sequential steps correctly, otherwise they had to start from the beginning of the sequence. Eight participants remained enrolled in the study for this stage. On average, the subjects could pick up 4.6 ± 0.9 blocks in each run in which the maximum number is 6. It took an average of 6.0 ± 0.5 seconds to finish each step (orange bar in Fig. 8a,b) and took 63.8 ± 5.1 seconds on average to move one block from the table onto the shelf. For those six subjects who participated in both the shelf-target grasp and fast-shelf-target grasp tasks, the performance under the two conditions is compared in Fig. 8a,b (light pink bar in the middle and green bar on the right side, respectively). In terms of the average blocks grasped in each run, performance was similar (5.1 ± 0.6 for normal speed vs 5.1 ± 0.5 for fast speed). However, it took on average 4.3 ± 0.7 seconds to finish each step in the fast-shelf-target grasp task compared to 6.0 ± 0.6 seconds for the shelf-grasp target task. Due to the faster speed of experiments in the fast-shelf-target grasp task (shorter intertrial interval, prefeedback periods, feedback periods and postfeedback periods at the same time), it took 40.6 ± 5.8 seconds on average (reduced about 36% of time compared to the previous normal speed one) to move one block from the table onto the shelf.

The distribution of PVC for moving targets from a table onto the shelf is displayed in Fig. 8c. The blocks on the x-y plane (table plane) show six possible positions at which the target might be placed, and the blocks on the x-z plane (shelf layers) show six possible positions at which the target might be moved to complete the task sequence. The color of the blocks shows the PVC of reaching to and then grasping/releasing the target. In general, the PVC of reaching and grasping/releasing for each location is higher than 71%. The lowest accuracy is located in the lower left corner (71%). The center portion of the plot shows the highest PVC on average (above 87%).

### Empirical chance level of virtual cursor task

We performed six sessions of resting state experiments to test the empirical chance level of the four and five virtual target experiments, respectively. Four subjects participated in the experiment and all subject data were pooled together for analysis. Each subject sat in front of a computer monitor and listened to relaxing and calming piano music. They were instructed to focus on the music, be relaxed, and stare at a center square on the screen. At the same time, the 2D virtual cursor task was running in the background but was only visible to the operator, hidden from the subject’s field of view. The average empirical chance level for the four and five target experiments were 25.3% (347 abort trials among 750 trials) and 22.5% (162 abort trials among 625 trials) respectively, which are close to the theoretical values of 25% and 20% for typical four/five target experimental paradigms without hovering time. The empirical numbers of target hit in each run by random chance were 1.8 ± 0.4 and 2.2 ± 0.5 for four and five virtual target experiments, respectively. Across all sessions from all participants, performance was significantly higher than expected by chance alone (see the green line for 2D cursor movement in Fig. 2).

## Discussion

Several challenges and opportunities exist for extending the field of BCI from virtual object control to physical devices and effectors in BCI research. To achieve such an extension, it is vital to study how human subjects interact with these physical devices through BCI control. Currently, BCIs driven by invasive technology have demonstrated control of prosthetic arms with high DOF to accomplish daily activities in a laboratory setting. These approaches utilize spike activity and local field potential signals from tens to hundreds of neurons in a local cortical area and decode these signals to control the position, orientation, velocity and/or force, among other parameters, of the prosthetic device by linear or nonlinear methods for neuronal ensembles^2^,^3^,^4^,^5^,^6^,^7^,^8^,^25^,^26^,^27^,^28^. Such invasive BCI technology is promising for mimicking the natural movement of the hand in paralyzed patients and can achieve relatively complex actions needed for daily life, such as reaching, grasping, and moving a water bottle towards the mouth. On the other hand, invasive BCIs are limited by the risks associated with surgical procedures and chronic implantation of electrodes in cortical areas, which highlights the need for the development of non-invasive BCI technology to meet the needs of different population groups. In this study, we demonstrate to accomplish reach-and-grasp/reach-and-release tasks in 3D space using a noninvasive EEG-based BCI by combination of two sequential low dimensional controls. A group of healthy human subjects participated in a series of longitudinal non-invasive EEG based BCI experiments. Each subject began with virtual cursor control and progressed to robotic arm control, with increasing complexity and dimensionality added over time. Throughout the experiments, we observed that most subjects exhibited improved performance over time in controlling both the virtual cursor and the robotic arm. While we used motor imagery paradigm and decoded the subject’s intention under the ERD/ERS framework as we used in our previous study to control a quadcopter^15^, the present study represents an entirely new investigation for human subjects to control a robotic arm for reaching, grasping and moving using noninvasive EEG signals. Recent work^18^ has explored the combination of motor imagination and other cognitive activities like alphabetical or numerical exercises to drive a robotic arm to complete reach task in a plane. Our work extends and explores the full possibility of reach and grasp of objects in a three-dimensional space, and furthermore more complex tasks close to the activities of daily living (ADL) like moving an object from table onto the shelf was designed and examined in multiple sessions. A successful trial consisted of touching the target, whether this was intentional or by chance, whereas our study design required subjects to hold the arm/cursor over the target to signal their intention. The current study has demonstrated that subjects are able to control a robotic arm to reach and grasp (with shared control) and move objects located in a constrained 3D space using a noninvasive BCI. The time scale of completing these intricate tasks was 20–60 seconds; with the exact duration depending on the complexity of the task and the performance of the subject. When moving objects from a table to a shelf with faster speed settings of robotic arm, we found that subjects could operate the arm with increased speed and shorter response time without sacrificing performance. To focus on subject learning and interactions with the BCI system, we employed a decoding method with minimal customization for each subject. In the future, optimization of the decoding algorithm for individual users and utilization of signals from source space through cortical imaging rather than from raw EEG signals^29^,^30^ could be of use for enhanced levels of robotic arm control and might reduce the time required to finish tasks. In addition, use of a high-speed robotic arm (which was not available in the present study) may also enhance the robotic arm control and further reduce the time required to finish the tasks.

### Sequential low dimensional control vs. fluid 3D control

In the current study, the combination of two sequential low dimensional control was utilized to realize the same function of fluid 3D control in 3D space. There are multiple ways to achieve reach-and-grasp in 3D space where the most efficient one is the direct 3D continuous control like human ourselves which our current approach does not belong to. To reach an object in 3D space, there could be a couple of combinations of sequential low dimensional control. One is the combination of three one dimensional sequential control which is the least efficient one while the other is the combination of a two dimensional control together with another sequential one dimensional control which is our case. This strategy aims to fully leverage the proficiency of two dimensional control for BCI subjects and encourage them to be fully engaged into the task due to the difficulty of the task. While at the same time a one dimensional control is designed for them in order to release the highly concentrated mental workload across all the time, to obtain higher accuracy and to enable lasting high performance duration (less frustration). Fluid 3D space continuous control of a robotic arm with high accuracy by noninvasive EEG requires at least three independent control signals (three pairs of motor strategies), which has not been shown yet. Previous research shows the promising capability of controlling a virtual cursor in 3D space^31^, however, the eight targets in the experiment was located in the corner of the virtual workspace and whether an target located at the random location of 3D workspace could be acquired with similar efficiency needs further investigation. Our sequential design is to balance the speed and accuracy to motivate subjects to engage into the experiments constantly. This sequential steps experimental design enabled the subjects, for the first time, to achieve reach and grasp in 3D space.

### ERD/ERS of virtual cursor control vs. robotic arm control

The event-related activity involved in virtual cursor control and robotic arm control showed high degrees of similarity. However, there was no strong ipisilateral ERS for unilateral hand motor imagination (right and left target) during the robotic arm control, and the bilateral ERS for the relax task (down target) during the robotic arm control was not as strong as its virtual cursor counterpart. This may be due to an inherently stronger resting state signal during robotic arm control, or a weaker ERS during the robotic arm control. There was no significant difference between the resting state signal for the 2D virtual cursor control and the robotic arm control, although we did observe a robust decrease of mu power at C3 and increase of power at C4 across tasks (Supplementary Table 1). This variation of resting state power, which was decreased for the robotic arm control compared to the virtual cursor control, may reflect the learning of BCI control and the modulation of subjects’ brain rhythms with increased task complexity. Thus, this eliminated the possibility of explaining the observed phenomena through an increased resting state signal for robotic arm control compared to virtual cursor control. We therefore speculate that the ERS decreased for the robotic arm control, which may seem to be counter-intuitive. We suppose that there might be smaller variation of ERD/ERS even though the ERS became smaller. Thus, the subjects still improve their brain rhythm control in general. The results in Supplementary Table 2 show that there is little difference in ERD between virtual cursor control and robotic arm control for the four different imagination tasks. However, it does display a significant difference in the ERS between virtual cursor control and robotic arm control across the different tasks.

Previous studies have shown a distributed cortical adaptation during learning of a BCI task where the modulated activity of a wide network decreases and focuses on a smaller network modulation when the user develops proficiency with BCI control^32^. Specifically, it has been previously shown that participants learned to increase the difference in frequency-specific power at the controlling electrode in up targets relative to down targets. This is not in accordance with ERS decreased for the robotic arm control and no difference in ERD was observed between virtual cursor control and robotic arm control when the users developed proficiency in the present study. First, results from the aforementioned study came from a relatively short learning period (two to three sessions over one to three days), while our findings revealed a relatively long-term learning curve for rhythmic modulation of cortical activity. Second, the previous study used the high-gamma band for modulation of the BCI control signal which is different from our upper mu rhythm control. These authors^32^ stated that mu-beta (12–30 Hz) band was also strongly task modulated but without the same changes of increase the difference in frequency-specific power at the controlling electrode, furthermore no conclusion could be obtained because of mu-beta rhythm was not the control signal in their study. The current mu band modulation during the mutiple sessions revealed that the mu-band ERD could be a stable control signal for the robotic arm control.

### Task design with hover period

We added the hover time as an additional level of control, such that the subject would need to confirm their intent to grasp an object. In this study, the subjects learned how to modulate their brain rhythm to reduce the speed of the cursor when the cursor approaches to and moves into the hover area in order to keep the cursor/robotic arm within the hover area. They could try more than once if the cursor/robotic arm shoot out of the hover area within two seconds. This hover period is not widely used with non-invasive BCIs but is commonly used with invasive BCIs^25^. The hover time is consistent with our daily experience of connecting with the environment. When we scan our surroundings for objects to interact with, passing through an available area or over a specific object does not necessarily mean we want to interact with it. When we stop and remain in a specific location for a certain amount of time, this usually indicates the intent to select this object. With this hold period, subjects have to learn how to control the cursor or the arm in a stable fashion for a certain amount of time. This training paradigm does increase the complexity of the task, as reported by participants’ verbal feedback, but it decreased the number of false positives in target selection that can often occur by chance and may increase the level of control of the subjects during the long-term learning period, which could be confirmed by further testing.

### Performance in the presence and absence of virtual cursor

The statistical analysis for the grasping of fixed targets in the presence or absence of the virtual cursor revealed that there was no difference between the two conditions, with the exception of the PVC for the five-target grasp task. There could be multiple reasons for this. Ideally the movement of the robotic arm should exactly comply with the movement of the cursor. This was true in most cases, but there was an occasional delay in the movement of the robotic arm if the brain rhythm generated a relatively large acceleration. Physical limitations restricted the robotic arm to lagging behind the control signal if the speed of the control signal exceeded the maximum speed limit of the robotic arm. Compared to the top-down view of the with-cursor tasks (the cursor represents the movement of the robotic arm), the perspective of the participant in the without-cursor tasks may have introduced some visual distortion regarding the position of the fingertips of the robotic arm and center of the blocks. For these reasons, subjects may have exerted more effort during the robotic arm paradigm compared to during the virtual cursor paradigm. This interpretation was corroborated by subjects’ verbal reporting. This could also explain why there was a marginal difference in PVC for the grasping of five targets (when the task became more difficult this effect was more apparent).

### The sustainability and variability of performance in long term

The high accuracies achieved by most of the subjects in the later sessions demonstrate that the ability to control one’s own brain rhythms by motor imagination can persist for long time periods (on a time scale of two to four months in our study). For each individual, the performance can vary day by day due to many factors, such as the daily mental status of each subject, slight alterations in electrode positions, the time of day during which subjects attend the experimental session, among other reasons^33^. However, despite these potential variations, subject performance remained high throughout the duration of the study. Unlike invasive BCIs, which can directly drive a prosthetic arm by decoding the neuronal activity in the motor cortex or posterior parietal cortex^2^,^3^,^26^,^27^,^28^, the non-invasive EEG based BCI utilized in this study translates the sensorimotor rhythms^34^ detected from bilateral motor areas to the activity of a single robotic arm. This is not intuitive to the subjects at the beginning of the experiments, but in later sessions subjects verbally report that control becomes more intuitive. During the learning of controlling a robotic arm across multiple sessions, subjects improved their ability to self-modulate specific brain rhythms in a focal motor area detected at a macroscopic scalp level which has implications for the design of noninvasive assistive and rehabilitation devices^35^.

During our experiments, multi-modal visual feedback was provided to the subjects that included activity on the computer monitor, the movement of the robotic arm and even the activity of the operator. Compared to other more controlled BCI paradigms, this complex environment is more similar to daily life. Importantly, our study revealed that these realistic environmental factors were not obstructive of subjects’ ability to learn and perform the BCI tasks. Subjects were able to control a robotic arm to complete reach-and-grasp tasks in three dimensions with up to over 80% accuracy for four or five-target grasp tasks. Looking towards the future, we plan to further develop and optimize technologies for non-invasive BCI control of prosthetic limbs in fluid 3D continuous control with high accuracy and increased speed. Ultimately, the goal of such systems will be to provide subject control of external prostheses in a non-invasive, naturalistic manner for aiding motor rehabilitation and control.

## Methods

### Experimental Setup

#### Subjects

18 healthy human subjects were recruited for the present study. Five of them dropped within the first three sessions due to their scheduling conflicts; the remaining 13 subjects (seven women; mean age, 27.3 y; range, 18–54) participated in 8–15 experiment sessions within an average of 81 ± 34 days. Each session took place on a different day, with one day to one week between sessions except for a few extreme cases. The length of duration was mainly due to scheduling need instead of the duration to learn the BCI skills. Data for these 13 subjects were analyzed for this study. All procedures and protocols were approved by the Institutional Review Board of the University of Minnesota. Informed consents were obtained from all of the subjects before they participated in the experiment. One of the subjects was left handed and all others were right handed. Eligibility and screening form is used to recruit healthy subjects within the age of 18–64 without traumatic brain injury or brain lesion and without any history of neurological deficit or neurodegenerative disorder in this study. The exclusion criteria were established before any subject was recruited to participate in the experiment. This study is registered with ClinicalTrials.gov, NCT02069938 (additional information about the clinical trial is available at https://clinicaltrials.gov/ct2/show/NCT02069938). All methods and research activities were performed in accordance with the guidelines and regulations.

#### Data Acquisition

EEG data were recorded by a 64-channel Neuroscan cap with SynAmps RT headbox and SynAmps RT amplifier (Neuroscan Inc, Charlotte, NC). The reference electrode was located on the vertex and the ground electrode was on the forehead. During the recording, the participants were seated in a comfortable chair and rested their hands on armrests. Each subject sat in front of a computer monitor at a distance of 90 cm. The robotic arm was mounted 50 cm to the right of the subject. All electrode impedances were maintained below 5 kΩ. The EEG signals were sampled at a rate of 1,000 Hz and bandpass-filtered in the range of 0.5–200 Hz. A notch filter of 60 Hz was applied to the raw EEG signals. A JACO arm (Kinova Robotics, Montreal, Canada), a seven DOF human-like robotic arm with three fingers, served as the BCI actuator and means of visual feedback for the subjects during the experiments. A Microsoft Kinect Motion Sensor was used to locate and send the position of a target to the computer. For the session of stage five, blocks were moved from table in front of the subjects to a three layer shelf of 5 inches × 19 inches × 19 inches.

### Task design for Brain-control Task

In the first stage, virtual cursor only, subjects were asked to complete only virtual cursor tasks for initial learning purposes. There were one to four sessions in this stage, depending on each subject’s performance. Each session consisted of four or five runs of one dimensional (1D) left vs. right cursor movement (1D,LR), four or five runs of 1D up vs. down (1D,UD) and four runs of two dimensional (2D) voluntary cursor movement in a plane if their 1D performance of (1D,LR) and (1D,UD) exceeded 80% accuracy on average in three consecutive runs for one session. Each run contained 25 trials of motor imagery tasks. The exact number of sessions and runs depended on the subject’s individual ability and availability. The subjects were instructed to imagine their left hand moving, their right hand moving, both hands moving or both hand relaxing to control left, right, up and down cursor movement, respectively (Fig. 1b).

In the second stage, four-target grasp, the subjects performed robotic arm control while a cursor was simultaneously moving on a computer monitor. Four foam blocks (size 4.5 cm × 5 cm × 10 cm) were placed in fixed positions on a flat table corresponding to the four target positions on the monitor. The hand of the robotic arm was controlled in end-effector velocity space. We defined a square workspace (size 32 cm × 32 cm) on the table to restrict where the blocks could be placed. The arm was also confined within this boundary to avoid collisions with the tabletop and participant. Each participant was required to complete two sessions of the four-target grasp on separate days. There were three runs of the 1D, LR control task, followed by three runs of the 1D,UD target grasping task and four runs of the four-target grasp task in each session. For the robotic arm to grasp an object on the table, a two-step task sequence of reach-and-grasp was employed to facilitate the participants’ ability to reach and grasp a block in 3D space. In the first step, a target object location was indicated to the subject on the monitor display. At this point, the subject would attempt to move the robotic arm within a horizontal plane to approach the center of the block at 17 cm above the block by performing the same 2D motor imagination tasks as for virtual cursor control. During the movement of the robotic arm, there was simultaneous cursor movement on the monitor. This was to represent the robotic arm’s hand position on the screen to make it clear that the physical target was approached and reached successfully; that is, the virtual cursor hit the target and changed colors from pink to yellow if the arm moved to the indicated block. A “hover area” was defined by a virtual cylindrical region centered above the target block with a radius of 3 cm. If the robotic arm maintained its position within the hover area for 2 seconds the trial was considered a successful hit and the task progressed to the second step of the sequence. In this step the subject was presented with a 1D UD robotic arm task to reach and grasp the block. Similar to the first step, if the arm was lowered down to the target and remained there within 2 cm of the center of the block for 2 seconds, the hand of the robotic arm would automatically grasp the target. Each subject participated in two sessions of this paradigm. The operators stood by and monitored the participant. The operators also placed or replaced the blocks as needed. The physical workspace containing the blocks was an area of 32 cm from left to right, 45 cm in depth, and 32 cm from front to back. The fingertips of the robotic arm returned to the center of the workspace at the beginning of each run.

In the third stage, five-target grasp, an additional block was placed in the center of the workspace, and was surrounded by the other four targets. This stage included the same reach-and-grasp sequence as stage 2. This stage was repeated for three sessions for each subject. Sessions were composed of one run of the 1D,LR cursor control task and one run of the 1D,UD cursor control task, followed by two runs of the 2D cursor control task and six runs of the five-target grasp task which is similar to four-target grasp task but with five blocks to grasp.

In the fourth stage, random-target grasp, the block was randomly placed in the pre-defined workspace by the operator. The participant was instructed to pick up the randomly positioned block using the same reach-and-grasp sequence as stages 2 and 3. The position of the randomly placed block was equally distributed among the four quadrants of the square workspace. The order of quadrant selection for block placement was randomly assigned. Once the subject successfully completed the reach-and-grasp task for the block, it would then be placed at another random location by the operator. If hovering in any place other than the target region for 2 seconds it would not proceed to the next step until reaching the maximum feedback duration (12 s) of the trial. For this stage, each subject was required to perform three sessions on separate days and sessions contained one run of both the 1D,LR and 1D,UD cursor control task, followed by three runs of the 2D cursor control task and five runs of the random-target grasp task.

In the fifth stage, shelf-target grasp, three blocks were placed on the table with fixed positions. Those positions were changed in each run. The participant was instructed to pick up the identified block and place it at a designated position within a three-layered shelf. The robotic arm started movement from the center of the cubic workspace and first move across the horizontal plane parallel to the table to select the block which was to be grasped. After the arm hovered above the specified block within the hover area for 2 seconds, the arm locked on the target and was then to move downward to grasp the target in the next step. These two steps were similar to the previous reach-and-grasp sequence. If the robotic arm grasped the target successfully it returned to the center, otherwise a new block location was selected and the procedure repeated until the subject successfully grasped a block from the table. When the subject successfully grasped a block on the table, the robotic arm moved back to the center and prepared for the vertical movement across the vertical plane parallel to the shelf. If the subject hovered over the specified position of the shelf for 2 seconds, the subject would be able to move forward and drop the block in the following step. This procedure also repeated until the subject successfully chose a position on the shelf and moved forward to drop the block at the specific position of the shelf. These two steps were named reach-and-release. In order to move a block successfully, the participants had to finish each of the four sequential steps correctly; otherwise they had to start from the beginning of the sequence of reach-and-grasp or reach-and-release. If the subject successfully placed a block onto the shelf, the empty space was filled with a new block (Paradigm ➎ in Fig. 1c). The positions for the targets in each run were fixed but varied across runs (see Supplementary Figure 6 for details). This paradigm was repeated during three sessions on separate days. Sessions contained one run of both 1D,LR and 1D,UD cursor control tasks, followed by three runs of the 2D cursor control task and five runs of the shelf-target grasp task.

Besides these five experiment stages, six of the subjects performed four extra sessions of the shelf-target task with decreased time periods between the different steps of the sequence. This additional stage was termed the fast-shelf-target task. The robotic arm was set to move with a constant speed of 8 cm/s for all of experiments in the above five stages. To test whether the subjects could operate the robotic arm with a higher speed by BCI control, the robotic arm was allowed to move with a maximum speed of 20 cm/s in the additional session of fast-shelf-target grasp task. In the fast-shelf-target grasp task session, the same task as in the shelf-target grasp task was repeated with a different parameter setting, where the prefeedback, feedback, postfeedback, and inter-trial interval duration were all decreased to about two thirds of the previous settings.

Finally, to further test the applicability of moving a block solely by the robotic arm in the absence of a cursor on the monitor, we repeated the four-target and five-target grasp tasks by removing the visual feedback of cursor movement from the subjects’ view. The subjects only received feedback from the movement of the robotic arm. These six subjects performed three sessions of the four-target and five-target grasp tasks in absence of the virtual cursor. Each of these sessions contained four runs of the four-target and two runs of five-target grasp tasks.

For each trial, there was an inter-trial interval that consisted of a black screen. This was followed by a “prefeedback” period indicating which target/block should be picked up, identified by a rectangular yellow bar on the monitor. After this was the “feedback” period in which the robotic arm moved according to the subject’s motor imagination toward the center of the specified block. The block was selected if the robotic arm remained in the hover area for 2 seconds (1 second for the fast-shelf-target grasp task). There was a maximum feedback duration (12 s) to let each trial end properly if the subject could neither hit or miss. Finally, if the target was selected, the hand of the robotic arm automatically opened or closed its fingers during the “postfeedback” period and was prepared for the next step’s grasping or releasing (Fig. 1d). During the fast-shelf-target task, the aforementioned segments of each trial were shortened to increase the speed of task progression and overall grasp sequence completion.

### Software and algorithm

BCI2000^36^ was used to control the movement of a virtual cursor and also to display the targets that indicated where the cursor should be moved to and which block should be selected. A custom C++ based program was used to control the movement of the robotic arm to track the position of the cursor. We acquired 62 channels of EEG signals; EEG channels C3 and C4 and surrounding channels over left and right motor cortex are utilized for online control. EEG activity from the controlling channels were spatially filtered by a small Laplacian filter^37^ and then fed into an autoregressive (AR) model to extract the power spectra features. The power activity in the upper mu frequency band over the left and right hemispheres were linearly mapped to the velocity of the virtual cursor or position of the robotic arm.

### Statistical analysis

Nonparametric statistical test, i.e. Wilcoxon signed-rank test is applied throughout the analysis of the results. Because the sample size in this study is relatively small, nonparametric statistical test is more appropriate. All of the significance tests are two sided and reported with the significance level of *α* = 0.05.

### Brain rhythm online calculation

The subjects learned to modulate their sensorimotor rhythm amplitude in the upper mu (10–14 Hz) frequency band over the left and right sensorimotor cortex to move the cursor and the robotic arm in one or two dimensions. An autoregressive (AR) model, as shown in Eq. (1), was used to estimate the amplitudes of sensorimotor rhythm:


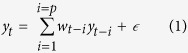


where *y*_*t*_ is the estimated signal at time *t, w*_*i*_ is the weight coefficient and 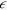 is the error of estimation. In the current study we applied the 16^th^ order AR model with a window length of 400 ms to calculate the online amplitude of mu rhythmic activity. The weight coefficients of which were estimated by the least-squares criteria.

### ERD/ERS quantification

The event related desynchronization (ERD) and event related synchronization (ERS) are brain oscillatory activity in diverse frequency bands. In this study we focus on the mu rhythmic activity which is modulated during participants’ motor imagination. The mu brain oscillatory activity during the experiment was externally paced by the appearance and disappearance of targets, or in other words, was time locked to the trial events. There are several methods to calculate the ERD/ERS time courses. In this paper, we used a bootstrap-based method^38^ to show a time-frequency map with significant changes of ERD or ERS for specific electrodes. In general, the calculation of ERD/ERS is performed by bandpass filtering the EEG signals, segmenting individual trials, detrending the trials, squaring the samples and subsequently averaging over trials and sample points. The procedures can be expressed as the following steps:


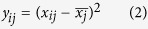



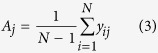



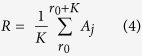



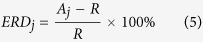


where *N* is the total number of trials, 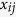 is the *j*th sample of the *i*th trial of the bandpass filtered EEG signals and 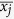 is the mean of the *j* th sample averaged over all trials. R is the average power in the reference period [*r*_*0*_, *r*_*0*_ + *K*], *r*_*0*_ is the starting time point of the reference period and K is the number of samples in the baseline reference period.

The above calculation provides the ERD/ERS values for each time point and each frequency bin of interest. In order to show those significant changes of ERD/ERS activity, we utilized the bootstrap resampling technique. This procedure is realized in the Biosig toolbox^39^.

### Data availability

All relevant data within the paper which are de-identified is available online at http://dx.doi.org/10.5061/dryad.nh109.

## Additional Information

**How to cite this article:** Meng, J. *et al*. Noninvasive Electroencephalogram Based Control of a Robotic Arm for Reach and Grasp Tasks. *Sci. Rep.* **6**, 38565; doi: 10.1038/srep38565 (2016).

**Publisher's note:** Springer Nature remains neutral with regard to jurisdictional claims in published maps and institutional affiliations.

## Supplementary Material

Supplementary Information

Supplementary Video 1

Supplementary Video 2

Supplementary Video 3
