# Author Correction: Noninvasive Electroencephalogram Based Control of a Robotic Arm for Reach and Grasp Tasks

**DOI:** 10.1038/s41598-020-63070-z

**Published:** 2020-04-15

**Authors:** Jianjun Meng, Shuying Zhang, Angeliki Beyko, Jaron Olsoe, Bryan Baxter, Bin He

**Affiliations:** 1 Department of Biomedical Engineering, University of Minnesota, Minneapolis, MN USA; 2 Institute for Engineering in Medicine, University of Minnesota, Minneapolis, MN USA

Correction to: *Scientific Reports* 10.1038/srep38565, published online 14 December 2016

This Article contains a typographical error in the spelling of the author Angeliki Beyko, which is incorrectly given as Angeliki Bekyo.

