# Supplementary material for: Noninvasive Electroencephalogram Based Control of a Robotic Arm for Reach and Grasp Tasks
Source: Sci Rep. 2016 Dec 14;6:38565. doi: 10.1038/srep38565 (PMC5155290; doi:10.1038/srep38565)
Supplement: Supplementary Information [file srep38565-s1.pdf]

Supplementary Materials

Noninvasive Electroencephalogram Based Control of a Robotic Arm for Reach and Grasp Tasks

Jianjun Meng[1], Shuying Zhang[1], Angeliki Bekyo[2], Jaron Olsoe[1], Bryan Baxter[1], and Bin He [1,2*]

1 Department of Biomedical Engineering, University of Minnesota

2 Institute for Engineering in Medicine, University of Minnesota

Supplementary Figures

Performance for grasping of fixed five targets task

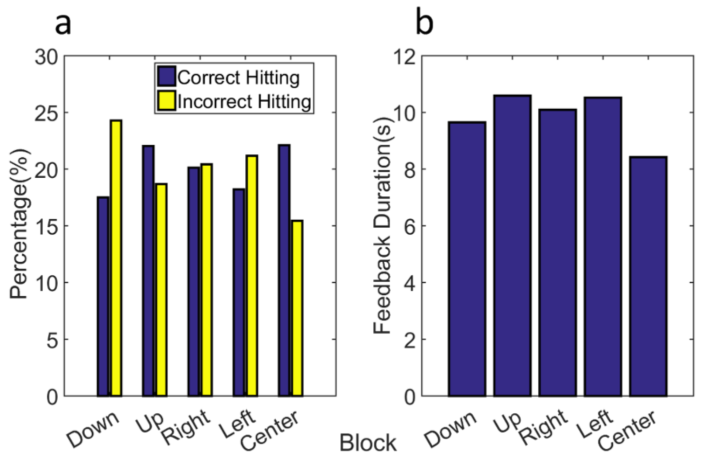

Supplementary Figure 1 Grasping distribution of the five-target grasp task. **a**) Average percentage of correct hitting and incorrect hitting for the different block locations across the three sessions. **b**) Average feedback duration for each block location including periods of both reaching to (hover) and moving downward to grasp the target.

In this third stage, a block was placed in the center of the restricted square area (**Fig. 1c** ❸). The initial position of the robotic arm was always located in the center of the square area as well to avoid bias toward any of the other blocks. Therefore, the arm was initially located above the center block and the participants had to move away from this block in two seconds if the specified block was not the center block. In **Supplementary Figure 1a**, the average distribution of correctly and incorrectly hitting targets for the five blocks for all subjects is displayed. Ideally, the accuracy should be balanced for each of the five targets (20% each); however, on average 22% of correct trials were up targets and center targets and about 17.5% down targets. This is in accordance with our observation that for most of the subjects it was easier to imagine movement of both hands (up targets) than to imagine relaxation of both hands (down targets). Accordingly, about 24% of incorrect trials were for the down targets, further indicating that the down target was harder for subjects to hit. It took subjects about 8.4s on average to grasp the center targets, which is shorter than that (about 10s) for other targets (see **Fig. S1 b**). This is

also congruent with the fact that the center target is right beneath the initial position of the robotic arm; theoretically it should take less time to grasp the center target if the subjects are able to hold the arm in the center of the target for 2 seconds.

EEG signals, topography and the arm movement trajectory for four-target grasp task

Supplementary Figure 2 displays a typical example of grasping one down target in the four-target grasp task, followed by the grasping of one up target immediately after. Another example of grasping one left target followed by the grasping of one right target is displayed in **Supplementary Figure 3.** The raw EEG signals were first spatially filtered using the surface Laplacian method. The signals were then bandpass filtered between 10 and 14 Hz. A time segment during 250s and 302s is displayed in **Fig. S2a** with the pink regions indicating time periods during each trial when the target was displayed. The robotic arm moved during the feedback period which is marked as the green region. Four topographies of upper mu power over a one second time window are shown in **Fig. S2b**. These one second time segments across four consecutive feedback periods are marked as small yellow bars in **Fig. S2a**. We can see that the brain rhythmic activity in the left and right sensorimotor cortex (around channel C3 and C4) are synchronized (ERS) when the subject imagined relax to drive the robotic arm go down to grasp or backward to reach the block and desynchronized (ERD) when the subject imagined bilateral hand movements to drive the robotic arm move forward to reach. The trajectories of the robotic arm during the feedback periods are illustrated in **Fig. S2c**. Each trajectory started at the center of the square workspace and moved in the direction of darker lines. The trajectories during the time window of topographies are marked in the yellow region. Similar subfigures for grasping a left block and a subsequent right block were shown in **Supplementary Figure 3**. We can also find that the ipsilateral motor cortex area synchronized and contralateral motor cortex desynchronized in the mu band when the subject imagined either left hand or right hand to move the robotic arm towards left or right direction, respectively (**See Fig. S3b**).

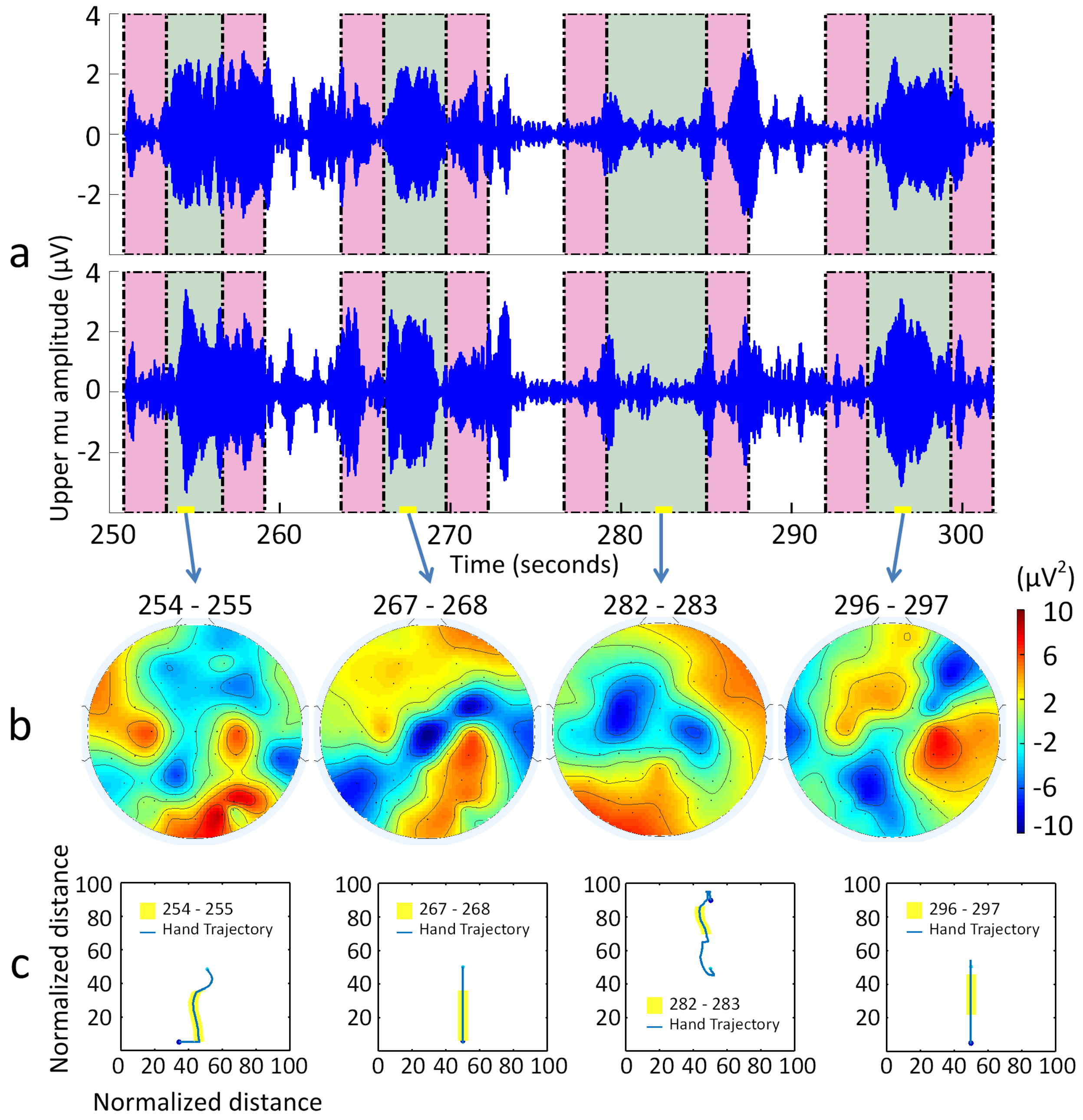

Supplementary Figure 2 EEG signals and scalp topographies associated with the up and down block grasping movement trajectories. **a**) The 10 -14 Hz bandpass filtered and Laplacian filtered EEG signals of channel C3 and C4 in the upper and lower rows, respectively. **b**) Normalized scalp topographies of power (12Hz) over one second time windows across the feedback periods of the up and down target grasping movements. **c)** Examplary robotic hand trajectories in the normalized coordinates of up and down target grasping for the four-target grasp task.

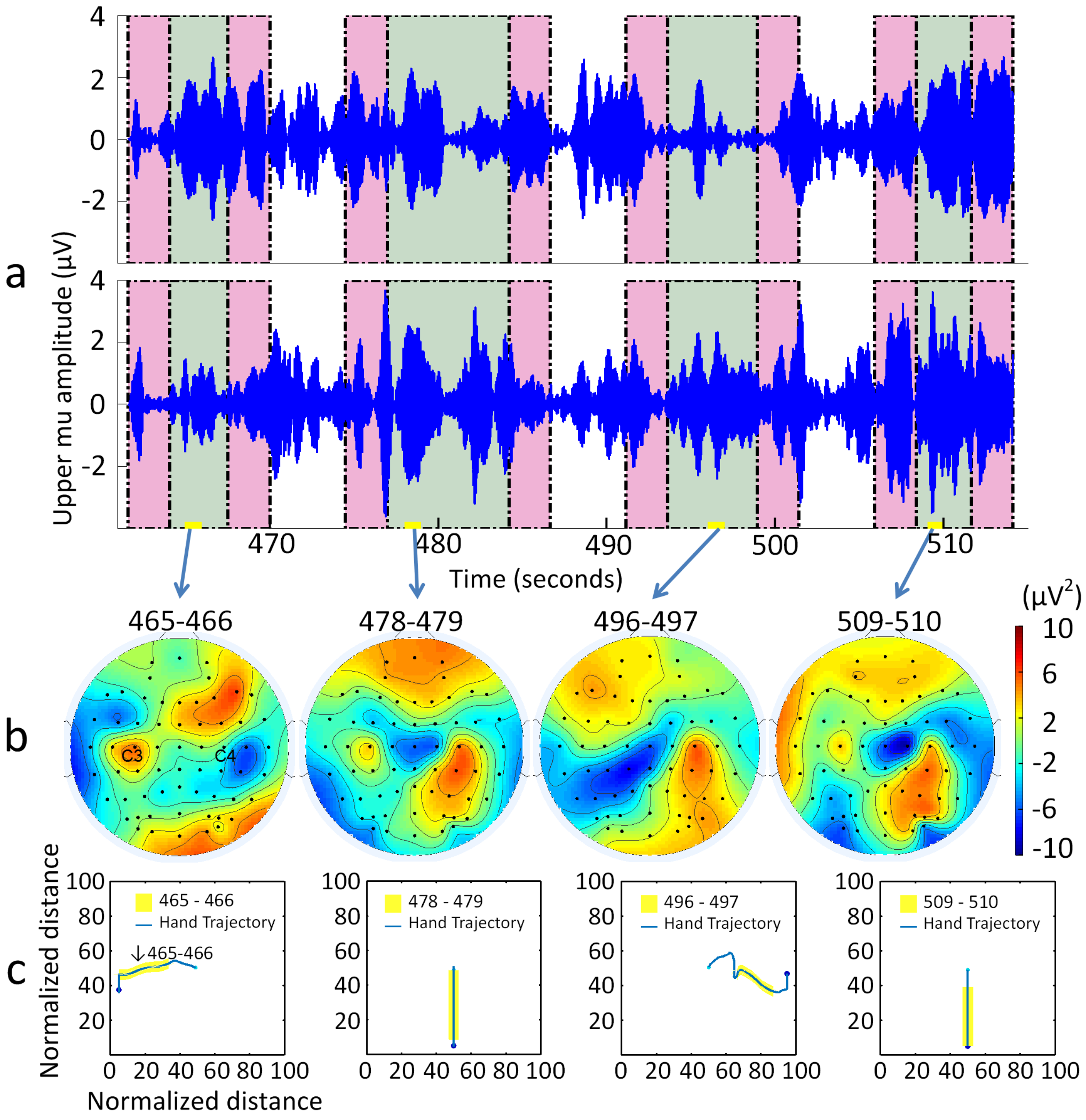

Supplementary Figure 3 EEG signals and scalp topographies associated with the left and right target grasping of movement trajectories. **a**) The 10 -14 Hz bandpass filtered and small Laplacian filtered EEG signals of channel C3 and C4 in the upper and lower rows, respectively. **b**) Normalized scalp topographies of power (12Hz) over one second time windows across the feedback periods of the left and right target grasping movements. **c**) Examplary hand trajectories in the normalized coordinates of left and right target grasping for the four-target grasp task.

Distributions of the random target locations for random-target grasp

Supplementary Figure 4 shows four examples of random target locations distributed by the operators for four different subjects. The x and y axes are normalized to 100. The green squares indicate those trials in which the subjects successfully grasped the block, while the crosses indicate those positions in which the subjects failed to grasp the block. Along the top and right sides of the figure, the histograms display the block distribution within the workspace. Due to the physical limitations of the robotic arm, we did not place targets in the left upper corner or lower right corner. The results in this paradigm indicate that subjects can grasp the target located randomly in the restricted square area without difficulties.

There were differences in accuracy for different movement directions. While performing the 'relax' task to move the robotic arm/cursor down, subjects had decreased accuracy compared to up/left/right. This may be because the definition of "relax" is ambiguous to subjects. The grasping distribution of the five fixed targets in **Supplementary Figure 1** also shows that there was a slight asymmetry ($p = 0.040$, $n = 13$, Wilcoxon signed-rank test) between the number of correct hits of up targets and down targets (22% vs. 17.5%). These results suggest that 'relax' may not be the ideal strategy of motor imagination for the down target. In the future, we would consider using foot motor imagination as an alternative (15).

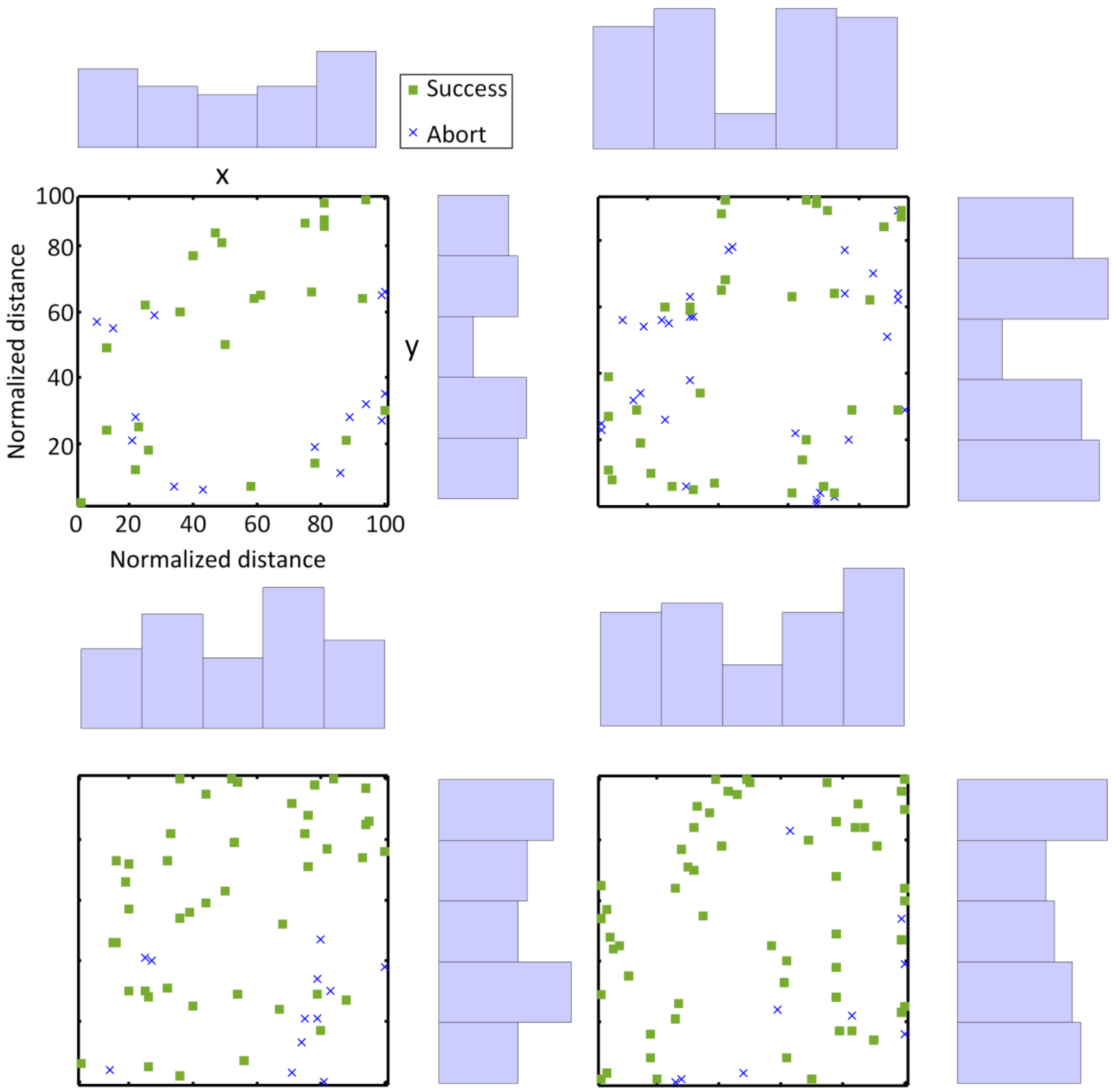

Supplementary Figure 4 Block distribution and successfully grasped targets for experiments of randomly located blocks. Four particular examples of block distribution for four subjects in four different sessions. The green squares indicate the positions where subjects successfully grasped the blocks, while the blue crosses display where the blocks were missed by the subjects.

The 3D space trajectory for shelf-target grasp task

Supplementary Figure 5 shows three particular examples of moving targets from the table onto the shelf for three subjects. The robotic arm started movement from the center of the cubic workspace and first move across the x-y horizontal plane to select the block which was to be grasped. After the arm hovered above the specified block within a predefined radius to the center of the target for more than

two seconds, the arm locked on the target. The arm was then to move downward to grasp the target in the next trial. Arm movement in all trials was controlled by motor imagination. If the arm grasped the target successfully it returned to the center, otherwise a new block location was selected and the procedure repeated until the subject successfully grasped a block from the table. When the subject successfully grasped a block on the table, the arm moved back to the center and prepared for the vertical movement across the x-z vertical plane in the next trial. If the subject hovered over the specified position on the shelf for more than 2 seconds, the subject would be able to move forward and drop the block in the following trial. This procedure would repeat until the subject successfully chose a position on the shelf and moved forward to drop the block at the specific position of the shelf.

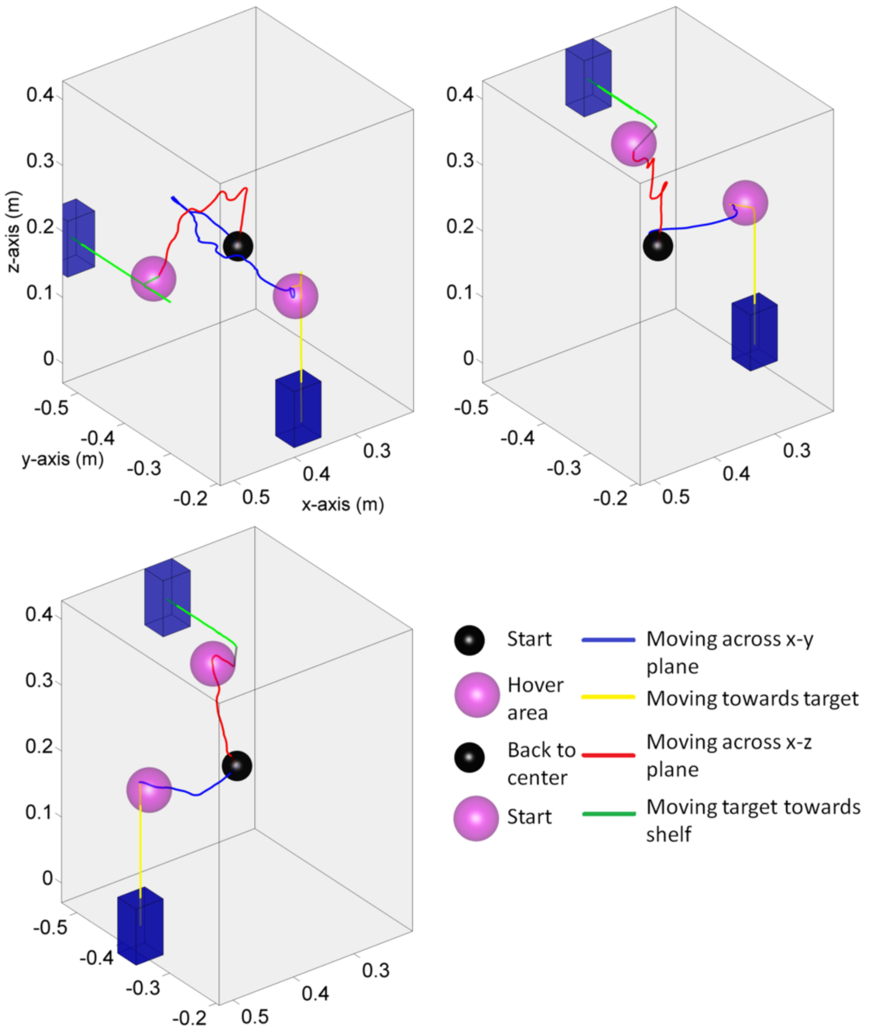

Supplementary Figure 5 Robotic hand trajectories for particular examples of three subjects. All of the hand trajectories followed the same sequence: the robotic hand started from the center of the cubic workspace (black sphere) and the subject moved the robotic hand from the start position to above a specific block (blue line) in the x-y plane. The robotic arm automatically opened its fingers if the hand remained in the hover area for more than a predefined period (2 seconds). Then the subject moved the robotic arm downward to grasp the targets on the table (yellow line) and moved back to the center (black sphere) automatically. The arm then moved across the x-z vertical plane to the hover area of an indicated position on the shelf where the block was to be dropped (red line), moved forward to the specified position on the shelf and dropped the target automatically onto the shelf (green line).

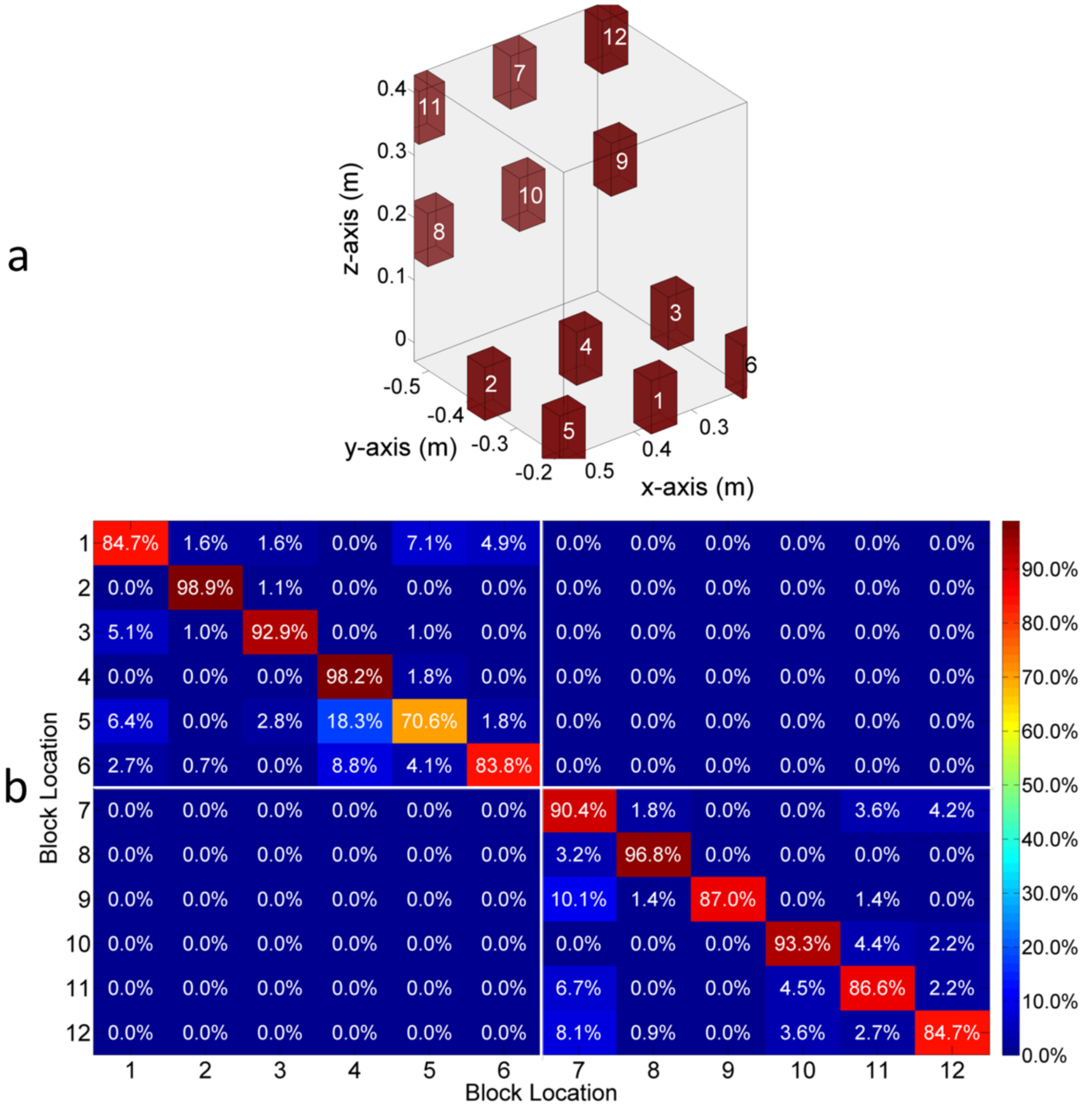

Supplementary Figure 6 Confusion matrix for the grasping of blocks on the table and releasing of blocks on the shelf. **a**) The six possible block locations on the table are labelled from one to six, and the six possible block locations on the shelf are labelled from seven to twelve. **b**) Confusion matrices for the grasping of targets on the table (block one to block six) and the releasing of targets on the shelf (block seven to block twelve).

Supplementary Figure 6a displays the six possible locations on the table and six possible locations on the shelf for the experiments in stage five. Note that three blocks were placed in three chosen positions on the table in each separate run (i.e. 1,2,3 for run 1 and the corresponding positions on the shelf were mirrored as 7,8,9; 4,5,6 for run 2 and the mirrored positions on the shelf were

10,11,12; 1,3,5 for run 3 and corresponding mirrored positions 7,9,11; 1,2,6 for run 4 and corresponding mirrored positions 7,8,12; 1, 5, 6 for run 5 and corresponding mirrored positions 7,11,12). Blocks were replaced to fill the empty space once a block was picked up and dropped to the shelf. **Supplementary Figure 6b** displays the confusion matrix for the grasping of blocks on the table from block one to block six and the releasing of blocks on the shelf from block seven to block twelve. The confusion matrix shows an average PVC of higher than 80% in each location except for position 5, located in the lower left corner of the workspace. When the specified block was in position 5 and an incorrect block was selected, it was most commonly block 4 (18.3%). This means that the robotic arm often remained at the center block because it could not move out of the hover area in the required 2 seconds, despite the subject trying to reach the block in the lower left corner (position 5).

Supplementary Tables

Right Hand

Channel \ Condition	Virtual Cursor	Robotic Control	Significance
C3	0.51±0.57	0.45±0.39	P = 0.89
C4	0.60±0.54	0.63±0.51	P= 0.68

Left Hand

Channel \ Condition	Virtual Cursor	Robotic Control	Significance
C3	0.57±0.66	0.41±0.37	P = 0.38
C4	0.56±0.48	0.50±0.41	P= 0.54

Both Hands

Channel \ Condition	Virtual Cursor	Robotic Control	Significance
C3	0.57±0.62	0.48±0.46	P = 0.54
C4	0.59±0.51	0.63±0.55	P= 0.64

Relax

Channel \ Condition	Virtual Cursor	Robotic Control	Significance
C3	0.56±0.65	0.47±0.33	P = 0.68
C4	0.59±0.52	0.62±0.37	P= 0.41

Supplementary Table 1: The power of bilateral mu rhythm at the resting state for four different imagination tasks (units μV^2). The power of mu rhythm at 12 Hz with a frequency bin of 3 Hz was calculated at the resting state and was used as the reference period (1.5 seconds prior to the appearance of targets). A Wilcoxon signed-rank test was applied to compare the difference of resting state powers between virtual cursor control and robotic arm control. Note that there was no significant difference in ERD/ERS between virtual cursor control and robotic arm control although we did observe a robust decrease of upper mu power at C3 and increase of power at C4 across tasks. This may imply the learning of BCI control and the modulation of subject's brain rhythms with increased task complexity.

Right Hand

Channel \ Condition	Virtual Cursor	Robotic Control	Significance
C3	-0.39±0.38	-0.51±0.20	P = 0.38
C4	0.57±0.72	0.02±0.28	P= 0.02

Left Hand

Channel \ Condition	Virtual Cursor	Robotic Control	Significance
C3	0.75±0.86	0.24±0.45	P = 0.04
C4	-0.40±0.48	-0.41±0.31	P= 0.91

Both Hands

Channel \ Condition	Virtual Cursor	Robotic Control	Significance
C3	-0.42±0.40	-0.55±0.20	P = 0.30
C4	-0.46±0.41	-0.46±0.33	P= 0.57

Relax

Channel \ Condition	Virtual Cursor	Robotic Control	Significance
C3	0.91±1.16	0.34±0.32	P = 0.02
C4	0.88±1.48	0.24±0.34	P= 0.06

Supplementary Table 2: The event related oscillations of bilateral mu rhythm during the feedback period for four different imagination tasks. The mean ERD/ERS of the mu rhythm at 12 Hz with a frequency bin of 3 Hz was calculated and averaged over the feedback period (marked in the red rectangular area of **Figure 3** and **Figure 4**). A Wilcoxon signed-rank test was applied to compare the difference of mean ERD/ERS during feedback period for four different imagination tasks between virtual cursor control and robotic arm control. Note that there is no difference in ERD between virtual cursor control and robotic arm control for the four different imagination tasks, but there exhibits a significant difference in the ERS between virtual cursor control and robotic arm control across the different tasks.

Supplementary Video Legends

Separate consents of video recording for research publications which were approved by the Institutional Review Board of the University of Minnesota were obtained from all of the subjects before recording their videos.

Supplementary Video S1

Examples of five-target grasp task. A subject move the robotic arm towards a right block among the five blocks on the table in 10 seconds by noninvasive EEG based control of a robotic arm. The subject moves the robotic arm to the top of specific block in the first step, where the specific target is indicated on the computer screen, and moves down towards the block to grasp it in the second step. Note that the subject has to keep the arm in the hover area for one second to make a successful selection. Followed by four subsplit screens, four subjects show similar grasping of specific blocks for the five-target grasp task in parallel.

Supplementary Video S2

Examples of random-target grasp task. A block is randomly placed on the table by the operator, where the position of the block is captured by a kinect and reflected on the computer screen, a subject use BCI to move the robotic arm towards the block in 15 seconds by a two step method. Followed by four subsplit screens, four subjects show similar grasping of specific blocks for the random-target grasp task in parallel. Note that the position of the randomly placed block is equally distributed among the four quadrants of the square workspace.

Supplementary Video S3

Examples of shelf-target grasp task. A subject moves the robotic arm to pick up a block from one of the three blocks on the table and move the block onto a specific position of a three layer white shelf in 30 seconds (several seconds of intertrial interval when the arm is still and the computer screen is blank are removed). The subject has to complete four steps to move one block from the table onto the

shelf in the shelf-target grasp task. Followed by four subsplit screens, four subjects show similar grasping of specific blocks on the table, moving and releasing of blocks on the shelf for the shelf-target grasp task in parallel.
